# Genome-Wide RADseq Reveals Genetic Differentiation of Wild and Cultured Populations of Large Yellow Croaker

**DOI:** 10.3390/genes14071508

**Published:** 2023-07-24

**Authors:** Kaifen Zhang, Yongdong Zhou, Weihua Song, Lihua Jiang, Xiaojun Yan

**Affiliations:** 1 Marine Science and Technology College, Zhejiang Ocean University, Zhoushan 316022, China; 2 Zhejiang Marine Fisheries Research Institute, Zhoushan 316021, China; 3 National Engineering Research Center of Marine Facilities Aquaculture, Zhejiang Ocean University, Zhoushan 316022, China

**Keywords:** *Larimichthys crocea*, RAD-seq, genetic structure, selective signal, genetic differentiation

## Abstract

*Larimichthys crocea* (also known as the large yellow croaker) is one of the most economically important marine fishes in China, and research on the ecology and genetics of this species is of immense significance. In this study, we performed restriction site-associated DNA sequencing (RAD-seq) of 54 individuals collected from four sites in China to analyze the genetic structure and diversity of large yellow croaker at the genome level. It revealed that the large yellow croaker populations in the Ningde and Zhoushan coastal waters can be clearly distinguished. Different genetic diversity indices were used to analyze the genetic diversity of the large yellow croaker, which showed that there was a differentiation trend between the wild and farmed populations in Ningde. Moreover, we identified genetically differentiated genomic regions between the populations. GO gene enrichment analysis identified genes that are related to fatty acid metabolism and growth. These findings enhance our understanding of genetic differentiation and adaptation to different living environments, providing a theoretical basis for the preservation and restoration of the genetic resources of the large yellow croaker.

## 1. Introduction

The large yellow croaker (*L. crocea*) is an economically important marine fish species distributed in the coastal waters and estuaries of the northwestern Pacific Ocean, including areas from central Vietnam to Korea and Japan, as well as the southern Yellow Sea, East China Sea, and South China Sea [1]. However, extensive overfishing in recent decades has severely depleted wild stocks of the large yellow croaker, which is on the brink of extinction. Since 1985, the artificial breeding and agricultural techniques of the large yellow croaker have rapidly developed. The annual production of *L. crocea* aquaculture in China has surpassed that of any other domesticated marine fish species [2]. In the 1980s, millions of *L. crocea* were successfully bred and released via artificial propagation. In the 1990s, the population genetic structure and genetic diversity of wild and farmed *L. crocea* in Fujian Province were analyzed using isozyme biochemical markers. The results revealed that both populations had relatively low genetic diversity levels [3]. For a long time, there have been studies on the development and application of molecular markers, such as AFLP and SSR, to analyze the genetic structure and diversity of different geographical populations of *L. crocea*. These studies have shown that the genetic diversity of domestically cultivated populations is generally lower compared to that of wild populations [4,5]. Moreover, previous molecular studies on *L. crocea* have relied primarily on mitochondrial DNA (mtDNA) sequences and offered limited resolution [6].

The reduced-representation genome sequencing (RRGS) technique can detect widely covered genetic markers throughout the whole genome, and it has advantages such as high throughput and low cost [7,8]. It has been widely used in selective sweep analysis and population genetic analysis [9,10]. In fish genomics research, the RAD-seq technique has been widely used for fish species differentiation, genetic maps, population genetics, and adaptive evolution [11,12,13,14]. Previously, genetic studies of the population structure, growth, and body shape-related traits in *L. crocea* using the ddRAD-seq technique have also been reported [15,16]. However, the molecular mechanisms underlying the differential response of *L. crocea* to living conditions, specifically between wild and farmed individuals, remain unknown.

Currently, there are differences in phenotypes between the farmed and wild populations of *L. crocea* [16], which could provide a good model for studying the adaptive evolution under different environmental conditions. In order to understand the population structure and the differences in adaptability between the natural and cultured environments, this study detected SNPs markers of the whole genome range of *L. crocea* populations based on the simplified genome sequencing technology (RAD-seq). Genetic diversity and population structure analyses were performed on three naturally wild populations captured from Zhoushan, Ningde, and Zhanjiang, as well as on a cultivated population. The identified candidate genes that differentiate the populations of *L. crocea* by using selective sweep analysis, and revealed their adaptive evolution mechanisms in different environments, providing scientific evidence for the recovery and protection of the resources of *L. crocea*.

## 2. Materials and Methods

### 2.1. Sample

**Figure 1:**
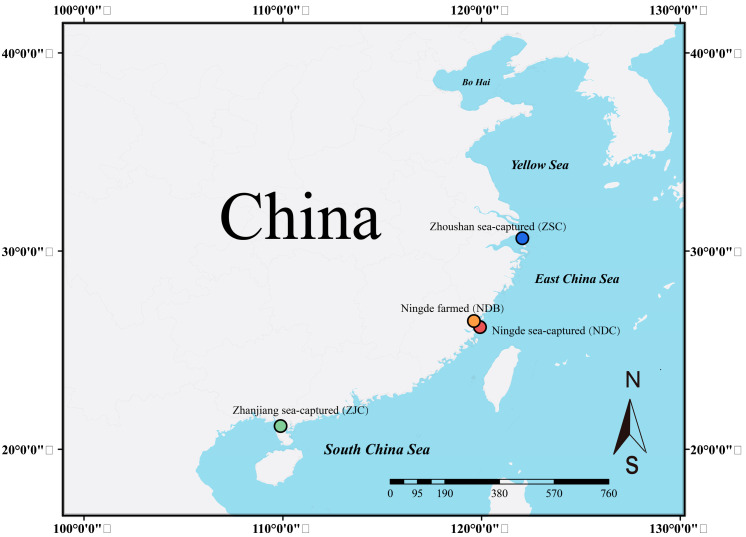
Geographic map and collection localities of the large yellow croaker in this study. ZSC = Zhoushan wild, NDC = Ningde wild, NDB = Ningde farmed, and ZJC = Zhanjiang wild.

We collected 54 individuals of *L. crocea* from three wild populations (ZSC, NDC, and ZJC) distributed throughout the coastal waters of China, and a farmed population (NDB) was collected from Ningde Fufa Aquaculture Co., Ltd. (Ningde, China). (Figure 1, Table S1). A piece of muscle tissue was obtained from each individual and preserved in 95% ethanol or frozen for DNA extraction.

### 2.2. Sequencing

High-quality DNA was used to build the restriction site-associated DNA (RAD) libraries. An equal amount of 1 μg of DNA from each individual was digested by two restriction enzymes (AvaII and MspI) and modified at the 3′ and 5′ ends, followed by sequencing adapter ligation to facilitate anchoring of the connecting products on the flowcell for bridge amplification. PCR amplification was performed to select appropriate fragments for library construction. Each sample was independently sequenced on the Illumina HiSeq X10 platform.

### 2.3. Variants Calling and Annotation

Raw sequence reads were processed to trim the adaptor and filter the low-quality reads using fastp [17]. The trimmed reads for the 54 samples were deposited in the National Center for Biotechnology Information (NCBI) BioProject database with accession number PRJNA985741. The cleaned reads were aligned to the *L. crocea* reference genome (GCA_000972845.2 version) using BWA [18]. The bam files were sorted, indexed, and filtered for redundant reads (MarkDuplicates) using SAMtools [19] to ensure accurate detection results. Subsequently, SNP variation detection was carried out using the HaplotypeCaller algorithm in GATK [20], with each sample generating its own gVCF before population joint genotyping. SNPs were filtered using GATK’s Variant Filtration with proper standards “QD < 2|| FS > 30.0”, and detected variants were then filtered SNP in clusters (more than 3 SNPs in a window of 35 bases). After filtering (--max-missing 0.9--maf 0.05), a high-quality set of variation loci was obtained. VCF tools [21] were used to count SNP density and total genetic diversity in each chromosome using a 100 kb sliding window.

SnpEff [22] was used to annotate the genomic distribution of variants and classify them into different categories (i.e., nonsynonymous, synonymous, UTR, upstream and downstream regions, intronic, and intergenic).

### 2.4. Population Diversity and Structure

We calculated basic statistics, including observed heterozygosity (HO), expected heterozygosity (HE), and inbreeding coefficient (*F*_IS_), for each population using PLINK [23]. We used ADMIXTURE [24] to estimate the genetic ancestry of each sample. This tool is based on the maximum likelihood estimates of the underlying admixture coefficients and ancestral allele frequencies. To improve the reliability of the results, the program was run 20 times by varying the values of K from 2 to 10. A cross-validation test was performed to determine the optimal value of K. In addition, fineRADstructure [25] was used to quantify the ancestry sources in each population and also explore the relationships between populations. FineRADstructure is a model-based Bayesian clustering approach that groups together individuals with high levels of shared co-ancestry. The high-resolution population structure inference is based on this co-ancestry matrix, which is used to cluster individuals according to the similarity of their RAD haplotypes. Principal component analysis (PCA) was performed using PLINK [23].

### 2.5. Linkage Disequilibrium Decay

In order to minimize the occurrence of unreliable genotypes, we used BCFtools [26] to eliminate individual genotypes with depths of ≤10 or ≥60. In addition, to minimize the potential confounding effects of population genetic structure, individuals with complex genetic backgrounds were filtered out. Moreover, SNPs deviating from Hardy–Weinberg equilibrium (*p* value < 0.001) were excluded, and we retained only SNPs with known genomic locations within a 1Mb distance. This approach is important as it helps prevent the overrepresentation of loci affected by selection hitchhiking.

PopLDdecay [27] was used to calculate the pairwise r^2^ between all pairs of SNPs (--minMAF 0.05--Miss0.1) located within 100kb of each other and plot the LD decay curve.

### 2.6. Analysis of Effective Population Sizes

The effective population size was calculated for each group using SNeP [28]. The parameter for the maximum number of SNPs per chromosome was set at 10,000.

### 2.7. Signatures of Selection

We used VCFtools [21] to estimate the nucleotide diversity (π) and the *F*_ST_ divergence statistic, with a window size of 100 kb and a step size of 10 kb for each population pair. We Z-transformed the distribution of *F*_ST_ and calculated the log value of θπ ratios [29]. The windows with the top 5% of values for the Z(*F*_ST_)and log_2_(π ratios) simultaneously served as the candidate outliers under strong selective sweeps. All outlier windows were assigned to their corresponding SNPs and genes. Selected genes enriched in gene ontology (GO) terms were determined using Metascape [30] (https://metascape.org/gp/index.html#/main/step1 (accessed on 17 February 2023)), with a significance threshold of *p* < 0.05 for enriched terms.

## 3. Results

### 3.1. Sequencing and SNP Discovery

After quality control of the sequencing data, the average number of RAD tags for each individual was 159,796, and an average coverage of 46× per locus was obtained by dd-RAD sequencing (Table S2). A total of 391,512 high-quality SNPs were used for subsequent analysis. Overall, the number of SNPs was positively correlated with the chromosome length, with chromosome 13 having the most SNPs (33,000) and chromosome 5 having the lowest (1289) (Figure S1, Table S3).

### 3.2. Genetic Diversity

We estimated basic population genetic parameters to determine the status of each population (Table 1). The observed heterozygosity (HO) ranged from 0.081 to 0.172 among the four populations, with an average HO that was lower than the average expected heterozygosity (HE). There was a significant difference between the observed heterozygosity (HO) of the wild population (NDC) and the cultured population (NDB) in Ningde. The range of nucleotide diversity among the populations was between 0.129 and 0.199. The ZSB populations had significantly lower HE and π values than those of the other populations. The genetic diversity was most abundant in NDC, with a nucleotide diversity (π) value of 0.199 and an observed heterozygosity (HO) of 0.172, followed by the wild population in Zhoushan (ZSC). We estimated the change in the effective population size over the past 1000 generations (Figure S2). All four populations showed decreasing trends in effective population sizes, suggesting that their genetic diversities remained at a low level. The inbreeding coefficients (*F*_IS_) of the populations were positive but not significantly different from zero. The ZSC group had the highest *F*_IS_ value (0.179) than the other group, indicating higher levels of inbreeding.

### 3.3. Population Structure and LD Decay

**Figure 2:**
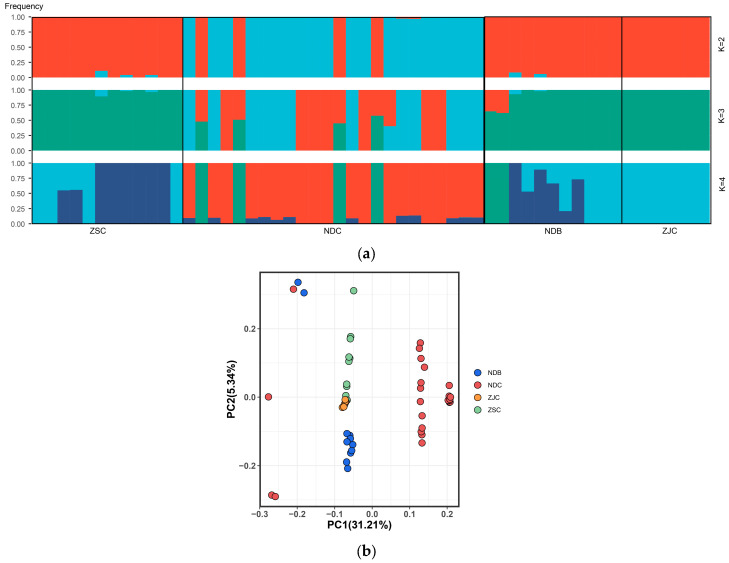

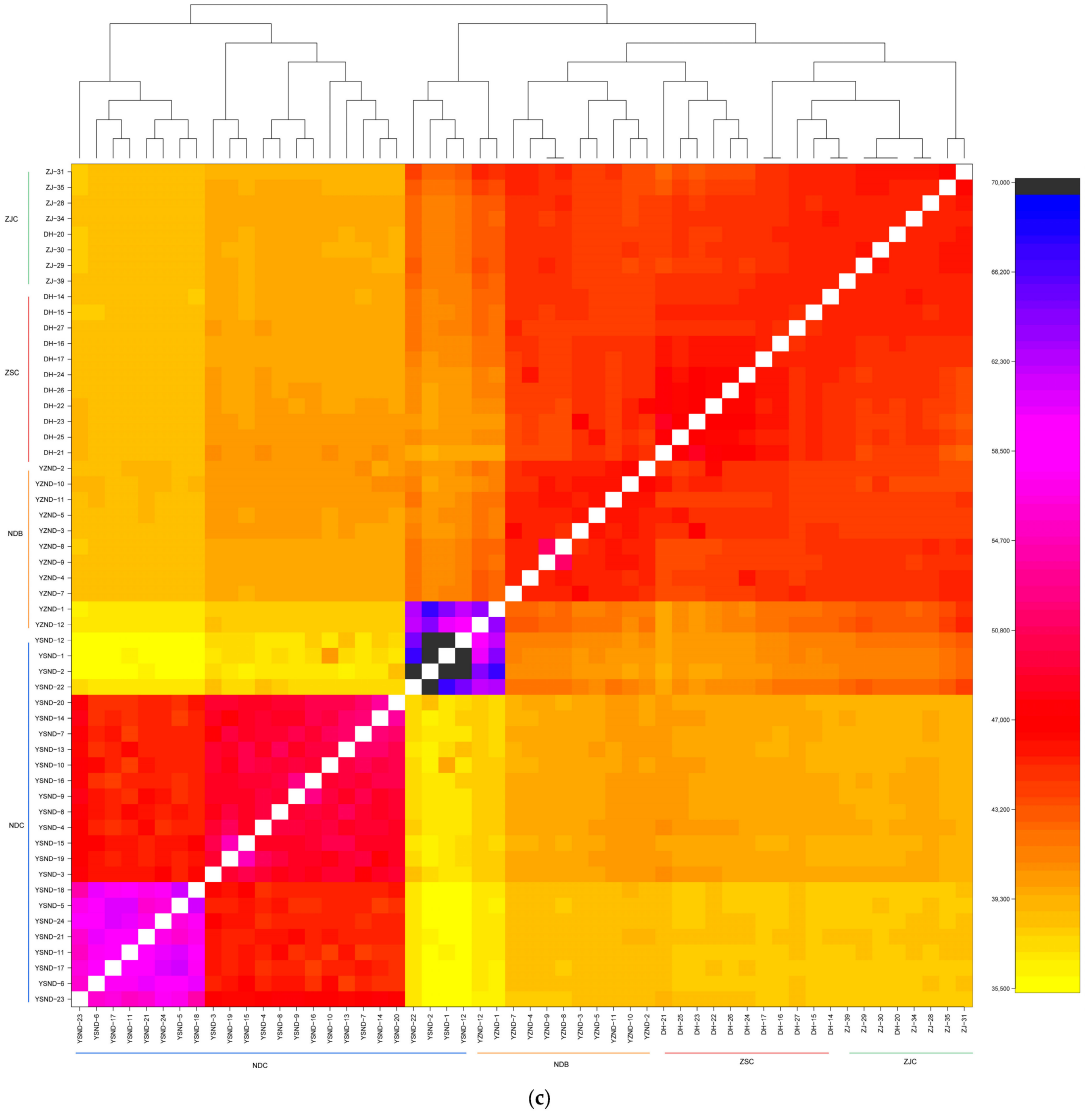

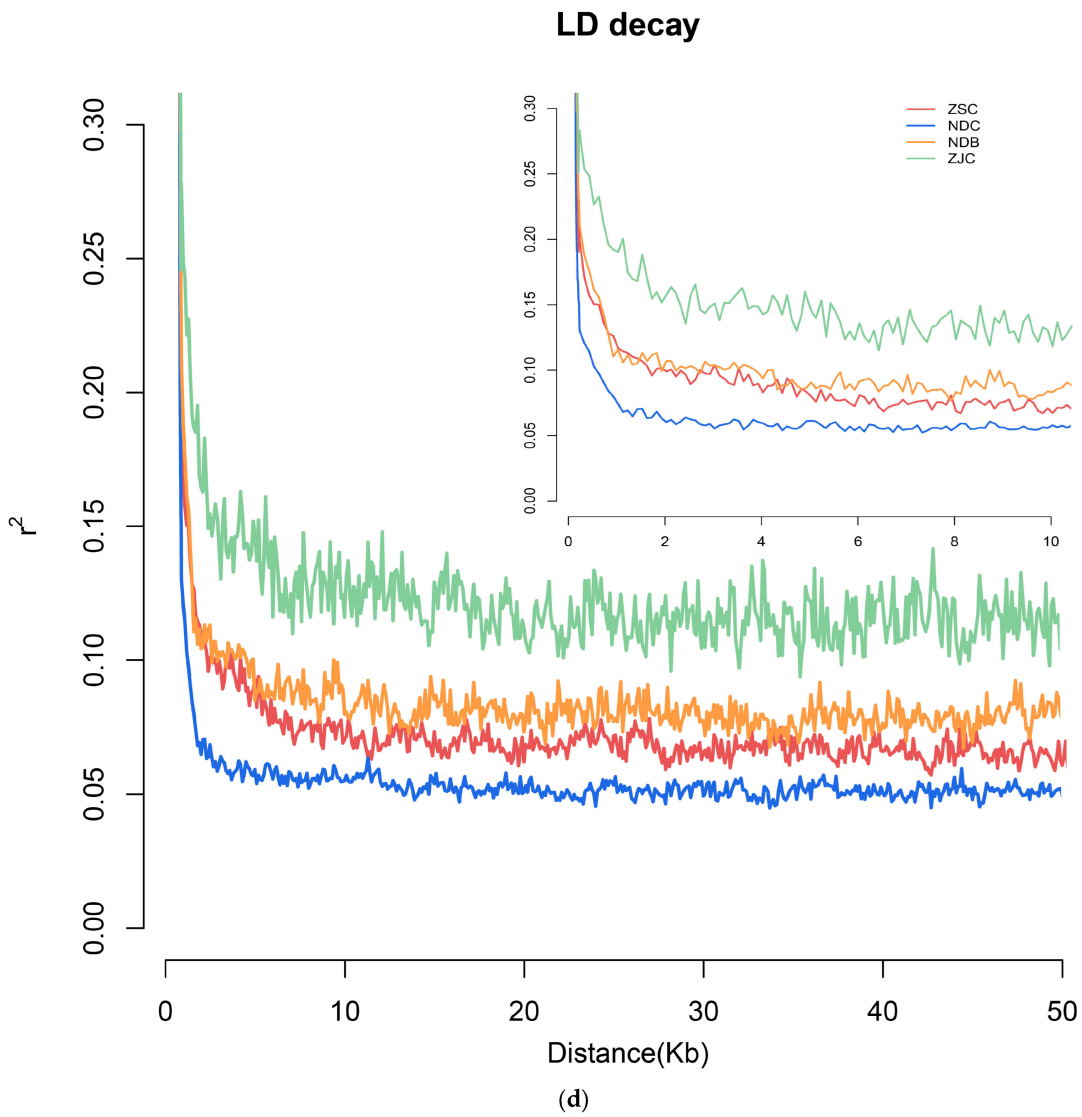
Population structure of the *L. crocea*. (**a**) Admixture analysis of four *L. crocea* populations. Length of each colored segment represents proportion of individual genomes inferred from ancestral populations (K = 2–4). (**b**) Principal components analysis of the *L. crocea*. (**c**) FineRADstructure analyses of the *L. crocea* population structure. (**d**) LD decay (r^2^) with distance for four populations.

The admixture analysis revealed clear evidence of clustering. Assuming clustering numbers (K values) of 2–4, the cross-validation error was lowest at K = 2, suggesting that the population genetic structure of our samples is best modeled by considering the admixture of the two genetic components (Figure 2a, Figures S3 and S4), and the 54 *L. crocea* individuals could be divided into two different groups.

A principal component analysis (PCA) recovered clusters similar to the admixture analyses. The first principal component (PC1) separated the NDC, which was consistent with the admixture result at K = 2. The second principal component (PC2) further separated the ZSB populations and was able to distinguish between the NDB and other populations (Figure 2b).

The most compelling detail about the population substructure was generated by the fineRADstructure method, and the resulting cladogram and co-ancestry matrix (Figure 2c) confirmed the results of the admixture and PCA, while also uncovering the presence of more subtle structuring. Two major clade clusters were also identified. The first clade was shown at the right of the cladogram and represented by a cluster of individuals in the top right of the co-ancestry matrix, which was further subdivided into three distinct clusters: the first comprising mainly individuals from ZJC and ZSC and the second comprising mostly farmed individuals from Ningde. A single population in the clade had a high level of intrapopulation co-ancestry. The remaining individuals shared little co-ancestry with the other individuals within the NDC. On the other hand, the second major clade was shown on the left of the cladogram, which was composed of wild individuals from Ningde, and the population showed high levels of shared ancestry.

The patterns of linkage disequilibrium (LD) indicated that the mean of the correlation coefficient values (r^2^) in the NDC group dropped rapidly at approximately 1 kb, while ZSC and NDB showed a relatively slower drop. In addition, the average pairwise correlation coefficient (r^2^) was higher in the ZJC than in other reference populations (Figure 2d).

### 3.4. Genome-Wide Selection Analysis

**Figure 3:**
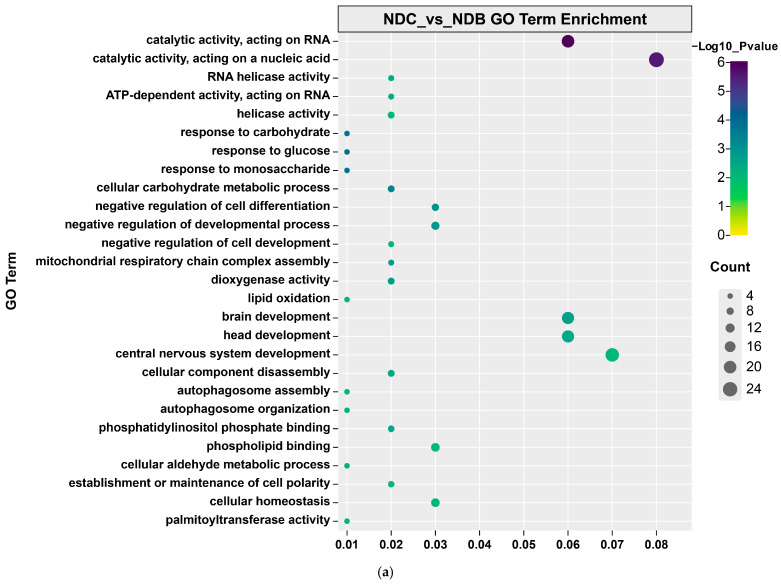

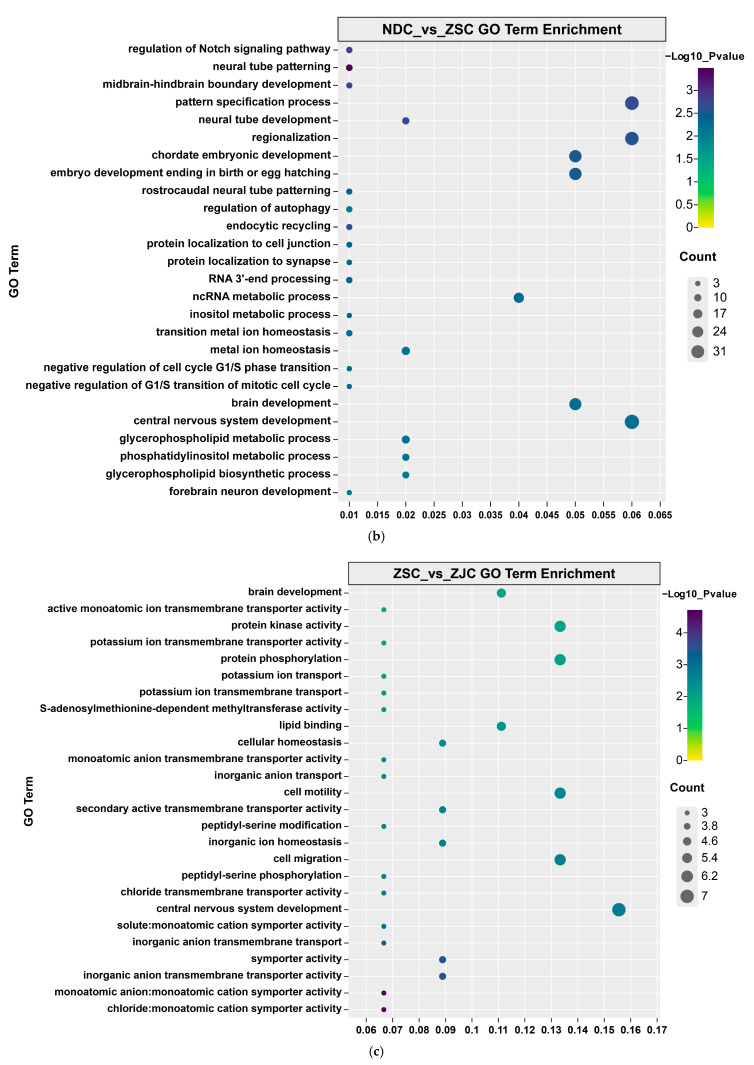

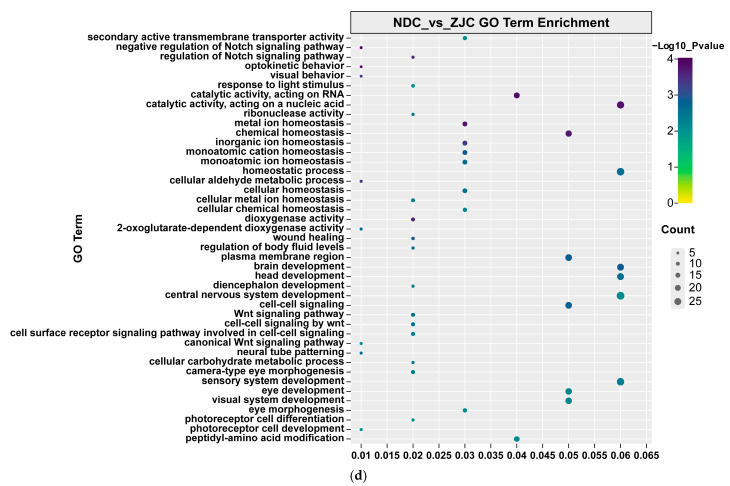
GO pathways in candidate genes. (**a**) GO Term Enrichment between the NDC and NDB; (**b**) GO Term Enrichment between the NDC and ZSC; (**c**) GO Term Enrichment between the ZSC and ZJC; (**d**) GO Term Enrichment between the NDC and ZJC.

We compared the genomes of *L. crocea* individuals to identify the genomic selection signals in different natural marine habitats and aquaculture environments. We identified genomic regions that may have undergone selective scanning by analyzing low levels of nucleotide diversity and conducting a combined analysis with *F*_ST_. Using the top 5% maximum Z(*F*_ST_) and log2(π ratio) values, the selected threshold was Z(*F*_ST_) ≥ 1.69 and log2(πNDC/πNDB) ≥ 1.75 (Figure S5a). A total of 96 highly differentially expressed genomic regions between NDC and NDB, which were annotated as 397 genes. The GO enrichment analysis results revealed that these genes were significantly enriched in biological processes such as thyroid hormone transmembrane transporter activity (*slco1d1*, *slc16a10*, and *slco1e1*), nucleosidases activity (*nt5c1bb*), and cellular aldehyde metabolism (*aldh3a2a*, *mlsl*, *aldh3a1*, and *idh1*) (Figure 3a, Table S4).

Similarly, a selective sweep analysis was conducted for large yellow croakers in the NDC and ZSC (Figure S5b). A total of 167 selected regions were identified, with annotations for 728 genes, which were significantly enriched in biological processes, such as inositol metabolism (*impa1*, *ppip5k1b*, and *bpnt2*), negative regulation of the cell cycle (*ccnd1*, *dlg1b*, and *fbxo31*), and glycerophospholipid metabolism (*pigm*, *efr3bb*, *socs2*, *pi4kb*, and *enpp2*) (Figure 3b, Table S4). For the *L. crocea* populations in ZSC and ZJC, a total of 19 selected regions were identified (Figure S5c), with annotations for 53 genes. GO enrichment analysis indicated that these genes were significantly enriched in biological processes, such as central nervous system development (*lhx1a*, *nr6a1a*, *nr5a1a*, *ttpa*, *tmx2b*, *xpr1b*, and *klf8*) and peptidyl-serine phosphorylation (*camkvb*, *vrk2*, and *csnk1g2a*) (Figure 3c, Table S4). In addition, a total of 148 selected regions were identified for large yellow croaker populations in the NDC and ZJC (Figure S5d), with annotations for 566 genes. GO enrichment analysis indicated that these genes were significantly enriched in biological processes, such as cell signaling *(slc12a7a*, *wnt8b*, *psen1*, *glra4b*, and *ptprn2*) and carbohydrate metabolism (*gys2*, *hk2*, *impa1*, *galk1*, *mlsl*, and *idh1*) (Figure 3d, Table S4).

## 4. Discussion

Previous research has indicated that the genetic structure of farmed *L. crocea* differs from that of wild populations. Domestication through farming may lead to changes in traits, such as body size, and exhibit clear signals of selection at the genomic level. However, studies based on early molecular markers have not thoroughly investigated the impact of farming domestication on the *L. crocea* genome and phenotype over the past few decades. Further research using whole-genome data may provide a more comprehensive understanding of the current status of the *L. crocea* genome and its relationship to phenotypic traits.

In this study, it was found that the genetic diversity level in the wild *L. crocea* in Zhoushan was relatively low. The unique and complex terrain of the Zhoushan nearshore area, along with the presence of numerous islands [31], may have led to limited gene flow between the populations of *L. crocea* in this area and those from other regions, leading to reduced wild resources and bottleneck effects due to genetic drift. Before the 1970s, *L. crocea* had obvious fishing grounds and a fishing season. In 1974, the fishing ground in Zhoushan was heavily fished, with the fishing intensity far exceeding the level that its resources could bear, and the natural resources of *L. crocea* in the Zhoushan fishing ground in the north of the East China Sea rapidly declined and nearly disappeared [32]. The high degree of genetic relatedness among individuals in the ZSC is likely due to the relatively limited recovery of wild population resources in the Zhoushan Sea area. Fishing data in recent years show that the marine fishing output of *L. crocea* in Zhejiang Province has increased, indicating that the wild population resources are gradually recovering [33].

Previous studies have shown that the genetic diversity of cultured populations of the large yellow croaker in Ningde, Fujian Province has significantly decreased compared to wild populations [4]. The results showed that the nucleotide diversity of the NDB was lower than that of the naturally captured population in the sea, which is consistent with the results of previous studies on the genetic diversity of large yellow croaker in the Ningde region using molecular marker techniques [4,34,35]. The relatively slower decay of LD in the farmed population was most likely caused by inbreeding within each strain, although the population structure and a possible increased rate of positive selection may also have contributed. However, the potential cause of the high linkage disequilibrium observed in the ZJC might be attributed to the low coverage depth.

The decrease in genetic diversity of the breeding population is attributed to the genetic bottleneck effect caused by the small effective number of parents and the accompanying genetic drift and inbreeding depression. After the successful artificial breeding and reproduction of the large yellow croaker in 1985, the industry rapidly developed, particularly in the coastal areas of Ningde in eastern Fujian. It is not surprising to find that the genetic background of the cultured large yellow croaker in Ningde is relatively homogeneous due to continuous artificial selection, and artificial selection has been broadly reported to possibly reduce genetic diversity [36,37]. While there is little population differentiation, a large amount of homozygote excess is observed within these populations. This finding suggests that there is a certain degree of inbreeding in large yellow croaker populations, which indicates a limited amount of allelic diversity. Moreover, the genetic diversity of the *L. crocea* populations in natural waters is also low due to scarce resources. This reflects the urgent need for genetic resource protection for this fish species.

Admixture analysis revealed that at K = 2, genetic differentiation was observed between the wild (NDC) and farmed populations (NDB) in Ningde, whereas some individuals showed signs of admixture, possibly due to gene flow. To further investigate the population structure, a fineRADstructure analysis was performed. The clustered co-ancestry heat map, generated with fineRADstructure using genome-wide SNPs, identified that the single population in our dataset, within NDC, had a high level of intrapopulation co-ancestry. However, several individuals in two populations (NDC and NDB) presented similar genetic ancestry, which suggested that they might have escaped farm fish with a higher frequency discharge rather than the natural-wild population [38]. In general, the results indicated that the NDC can be differentiated from the other three populations, including the NDB.

There are significant differences between wild and farmed populations of large yellow croaker, such as body length, body shape, and the content of polyunsaturated fatty acids, which may be caused by potential SNP loci that drive their different traits. The farmed population in the Ningde region primarily comprises descendants of about 30 parent fish that were caught in the same sea area back in 1985–1986 and subsequently bred artificially [38]. In this study, the initial breeding of the farmed population and the wild population both originated from the same sea area. As domesticated environments are distinct from natural marine areas, it is speculated that different populations have caused SNP variations over time due to their adaptations to different living conditions.

Gene ontology analysis revealed genomic adaptation to the cultivated environment via selective scanning. These results suggest that the population of the large yellow croaker has genomic features that adapt to different survival environments. This study has shown that the significantly enriched pathway was related to the transmembrane transport of thyroid hormones. Thyroid hormones regulate many important functions in teleost fish, such as growth, skin pigmentation, morphogenesis, and metamorphosis, during early development [39]. The *slco1d1* is responsible for the ingestion of steroid hormone conjugations into liver cells, and changes in its expression may affect the amount of hormone conjugations, which may affect the embryonic development and morphological behavior of zebrafish [40].

Moreover, differences were observed in genes involved in early embryonic development. *Ddx4* is an important regulatory factor for determining germ cell development and is the first gene involved in primitive germ cell development [41]. *Ddx4* is widely used as a DNA molecular marker for fish gonadal development, gender determination, and differentiation [42,43,44,45,46]. *Brd3a* is necessary for the differentiation of embryonic stem cells into blood platelets during early embryonic development [47]. We identified *npffr1* genes located on chromosome 3 that have a strong selection signal. *Npffr1* is involved in the control of feeding behavior in vertebrates and invertebrates [48,49]. Therefore, it is hypothesized that the *npffr1* gene may be associated with growth and weight in the large yellow croaker. This may be evidence of artificial selection affecting the developmental processes of the farmed large yellow croaker, which grows faster and tends to mature earlier than the wild population.

Fatty acid desaturase 2 (*fads2*) is a key enzyme in the biosynthesis of long-chain polyunsaturated fatty acids (LC-PUFA) in vertebrates, which is considered the rate-limiting step and is commonly used as an indicator of LC-PUFA biosynthesis [50]. The differences in LC-PUFA biosynthesis among different species are the results of differences in *fads2* activity [51]. A comparison of the fatty acid composition in muscle between the farmed and wild large yellow croaker revealed that (*n* − 3)/(*n* − 6) LC-PUFA ratio is higher in the wild population [52]. We speculate that the difference in LC-PUFA content between wild and farmed fish may be related to this gene, but further verification is needed. Additionally, we also discovered GO terms related to the mitochondrial respiratory chain complex in farmed populations, which may affect cellular respiration and energy metabolism. Compared to the aquaculture environment, natural marine environments may have limited food, and capturing large yellow croakers requires more energy to obtain prey. Soybean meal was widely fed in the large yellow croaker farming industry [53]; as a result, there are notable variations in genes linked to energy metabolism, such as carbohydrate derivative metabolic processes and fatty acid metabolic processes, and the Ningde wild and farmed populations.

## Figures and Tables

**Table 1:** Genetic diversity of the *L. crocea* populations based on the SNP.

Population ID	Observed Heterozygosity	Expected Heterozygosity	Nucleotide Diversity (π)	Inbreeding Coefficients *F*_IS_
ZSC	0.097	0.142	0.155	0.179
NDC	0.172	0.222	0.199	0.106
NDB	0.093	0.122	0.129	0.107
ZJC	0.081	0.123	0.136	0.133

## Supplementary Materials

The following supporting information can be downloaded at: https://www.mdpi.com/article/10.3390/genes14071508/s1, Figure S1. Density distribution of SNPs on different chromosomes. The horizontal lines represent the total length of the chromosomes, and different colors or grayscales represent different SNP densities. Figure S2. Effective population sizes of four populations over the past 1000 generations. All four populations showed decreasing trends. Figure S3: The cross-validation error distribution according to the number of clusters (K) by ADMIXTURE; Figure S4: Admixture analysis of four populations. Length of each colored segment represents proportion of individual genomes inferred from ancestral populations (K = 2–10). Figure S5: Genomic regions with strong selective signals in populations. Table S1: Detailed information of sampling; Table S2: Sequencing outputs; Table S3: Annotation and classification of identified single nucleotide polymorphism (SNPs); Table S4: Enriched functional categories.
