# Supplementary material for: Genome-Wide RADseq Reveals Genetic Differentiation of Wild and Cultured Populations of Large Yellow Croaker
Source: Genes (Basel). 2023 Jul 24;14(7):1508. doi: 10.3390/genes14071508 (PMC10379082; doi:10.3390/genes14071508)
Supplement: Supplementary file 1 [file genes-14-01508-s001.zip › Suppl figure.pdf]

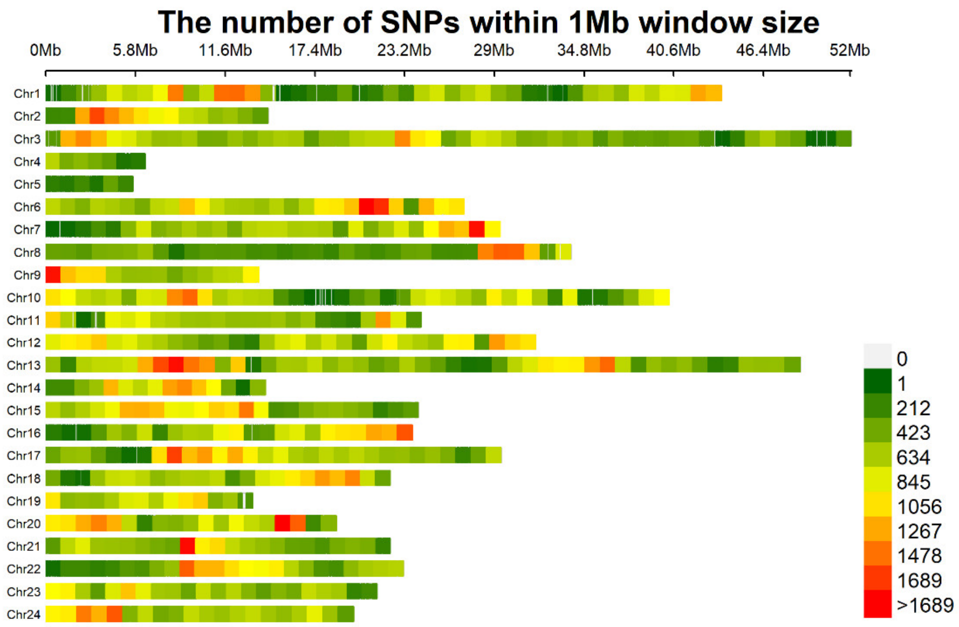

Figure S1. Density distribution of SNPs on different chromosomes. The horizontal lines represent the total length of the chromosomes, and different colors or grayscales represent different SNP densities.

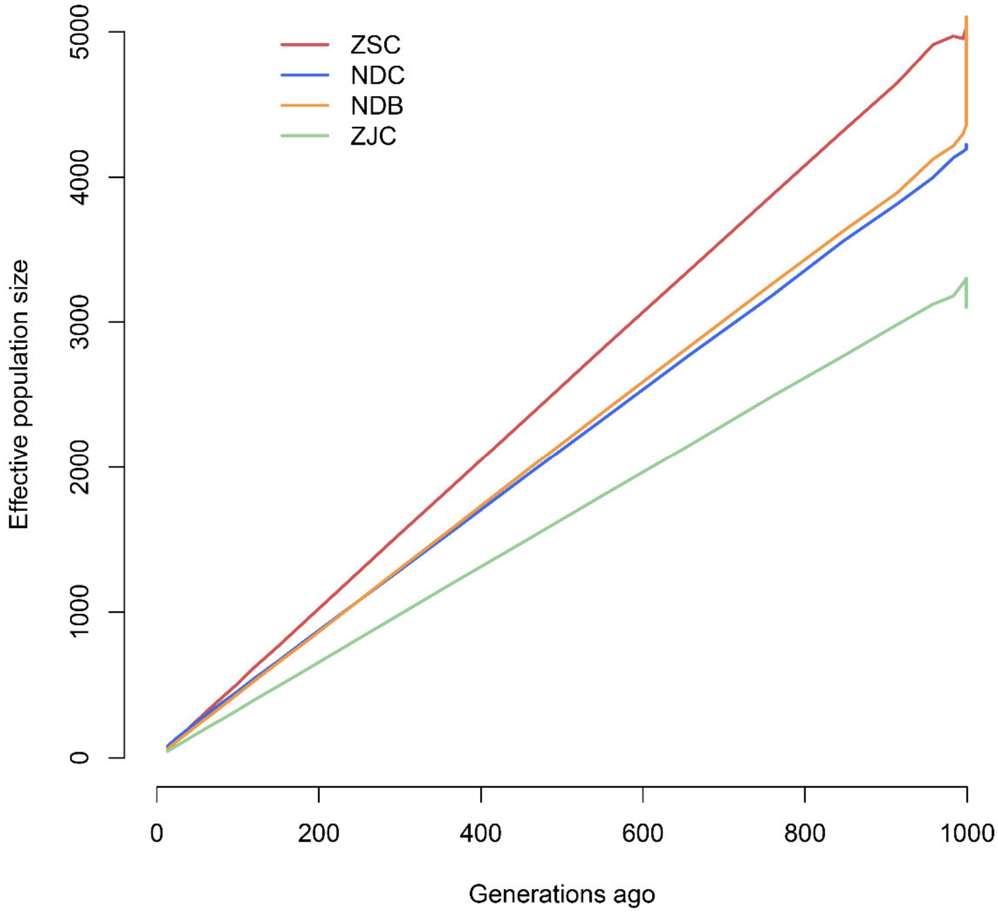

Figure S2. Effective population sizes of four populations over the past 1000 generations. All of the four populations showed decreasing trends.

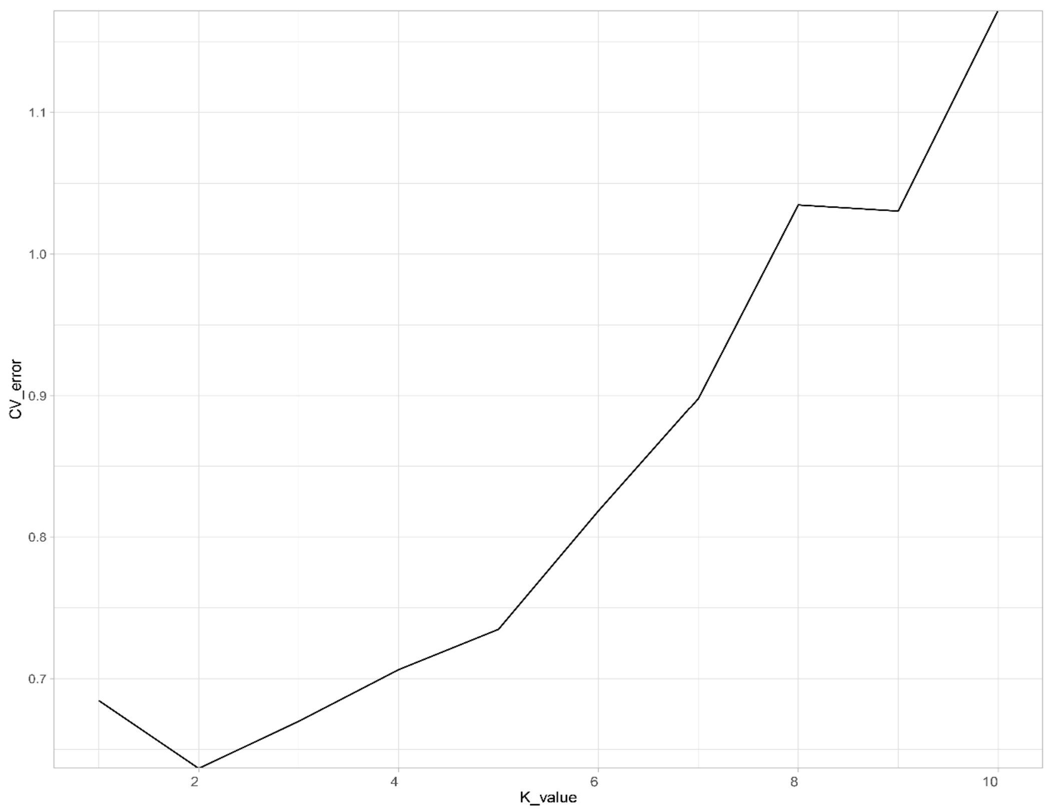

Figure S3. The Cross-Validation error distribution according to the number of clusters (K) by ADMIXTURE.

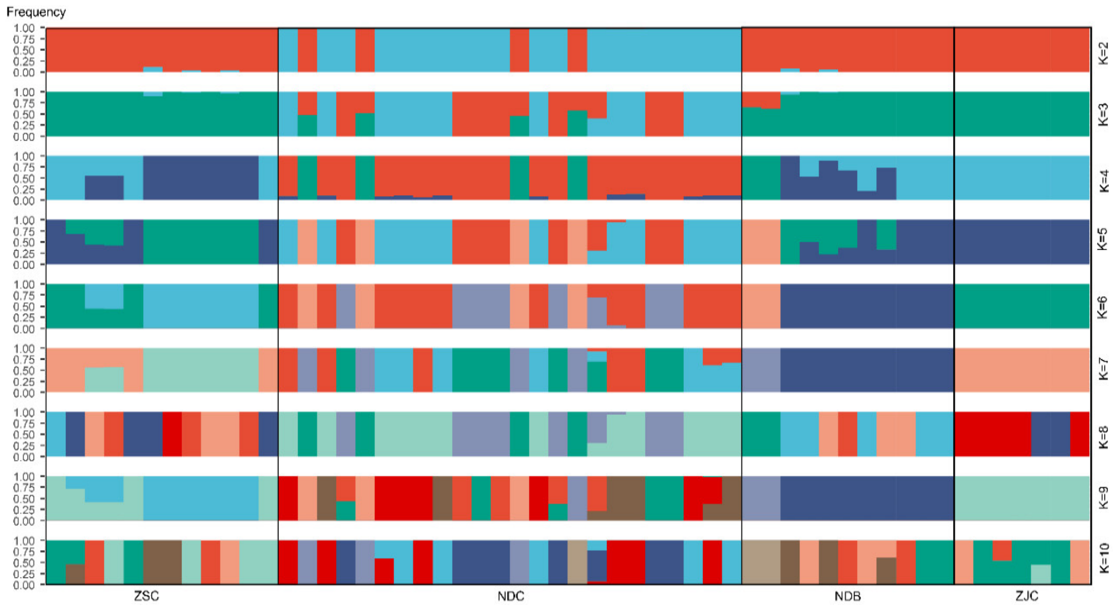

Figure S4. Admixture analysis of four L. crocea populations. Length of each colored segment represents proportion of individual genome inferred from ancestral populations (K=2-10).

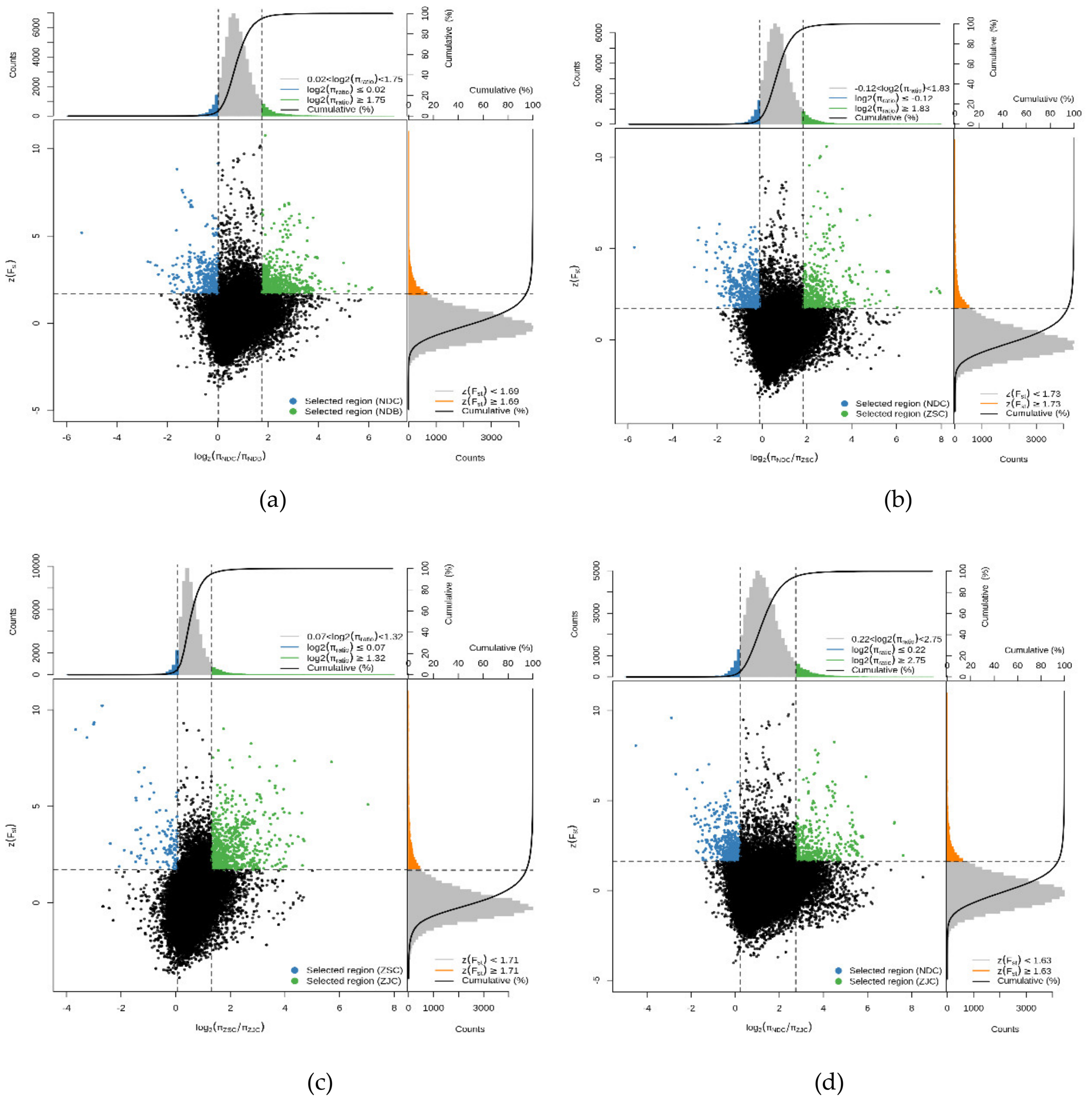

Figure S5. Genomic regions with strong selective signals in populations of *L. crocea*. (**a**) Distribution of log2(θπ ratios) and $Z(F_{ST})$ values calculated in 100 kb sliding windows with 10 kb increments between NDC/NDB populations. Data points in green (corresponding to top 5% of empirical log2[π ratio] distributions with values >1.75 and $Z(F_{ST})$ distributions with values >1.69) are genomic regions under selection in NDB population; (**b**) Distribution of log2(θπ ratios) and $Z(F_{ST})$ values calculated in 100 kb sliding windows with 10 kb increments between NDC/ZSC populations; (**c**) Distribution of log2(π ratios) and $Z(F_{ST})$ values calculated in 100 kb sliding windows with 10 kb increments between ZSC/ZJC populations; (**d**) Distribution of log2(θπ ratios) and $Z(F_{ST})$ values calculated in 100 kb sliding windows with 10 kb increments between NDC/ZJC populations.
